# Novel Insights into *Aspergillus fumigatus* Pathogenesis and Host Response from State-of-the-Art Imaging of Host–Pathogen Interactions during Infection

**DOI:** 10.3390/jof8030264

**Published:** 2022-03-04

**Authors:** Sébastien C. Ortiz, Katie Pennington, Darren D. Thomson, Margherita Bertuzzi

**Affiliations:** 1 Manchester Academic Health Science Centre, Core Technology Facility, Manchester Fungal Infection Group, Faculty of Biology, Medicine and Health, The University of Manchester, Grafton Street, Manchester M13 9NT, UK; sebastien.ortiz@manchester.ac.uk (S.C.O.); katie.pennington1@btinternet.com (K.P.); 2 Medical Research Council Centre for Medical Mycology, University of Exeter, Geoffrey Pope Building, Stocker Road, Exeter EX4 4QD, UK; d.d.thomson@exeter.ac.uk

**Keywords:** *Aspergillus fumigatus*, host–pathogen interaction, advanced imaging technologies, lung deposition, fungal germination, host response, mucosal immunity, zebrafish, infection imaging

## Abstract

*Aspergillus fumigatus* spores initiate more than 3,000,000 chronic and 300,000 invasive diseases annually, worldwide. Depending on the immune status of the host, inhalation of these spores can lead to a broad spectrum of disease, including invasive aspergillosis, which carries a 50% mortality rate overall; however, this mortality rate increases substantially if the infection is caused by azole-resistant strains or diagnosis is delayed or missed. Increasing resistance to existing antifungal treatments is becoming a major concern; for example, resistance to azoles (the first-line available oral drug against *Aspergillus* species) has risen by 40% since 2006. Despite high morbidity and mortality, the lack of an in-depth understanding of *A. fumigatus* pathogenesis and host response has hampered the development of novel therapeutic strategies for the clinical management of fungal infections. Recent advances in sample preparation, infection models and imaging techniques applied in vivo have addressed important gaps in fungal research, whilst questioning existing paradigms. This review highlights the successes and further potential of these recent technologies in understanding the host–pathogen interactions that lead to aspergillosis.

## 1. Introduction

*Aspergillus fumigatus* spores are a major component of airborne particulate matter (1–100/m^3^) [1] and initiate more than 3,000,000 chronic and 300,000 invasive diseases per annum, globally [2,3]. Depending on the host’s immune status, inhalation of *A. fumigatus* spores can lead to a broad spectrum of disease, including invasive aspergillosis (IA; affecting in particular patients with chronic obstructive pulmonary disease, COPD) [4,5], chronic pulmonary aspergillosis (CPA) and allergic bronchopulmonary aspergillosis (ABPA; affecting more than 4 million asthma and cystic fibrosis, CF, sufferers) [6]. IA carries a 50% mortality rate overall; however, mortality rates approach 100% if diagnosis is delayed or missed and >75% in certain cohorts of patients, notably those with COPD. Currently, mortality increases substantially for infections with azole-resistant isolates, and resistance to azoles (the first-line available oral drugs against *Aspergillus* spp.) has risen by 40% since 2006, especially due to systemic azole usage for agricultural purposes [7,8,9]. 

**Figure 1:**
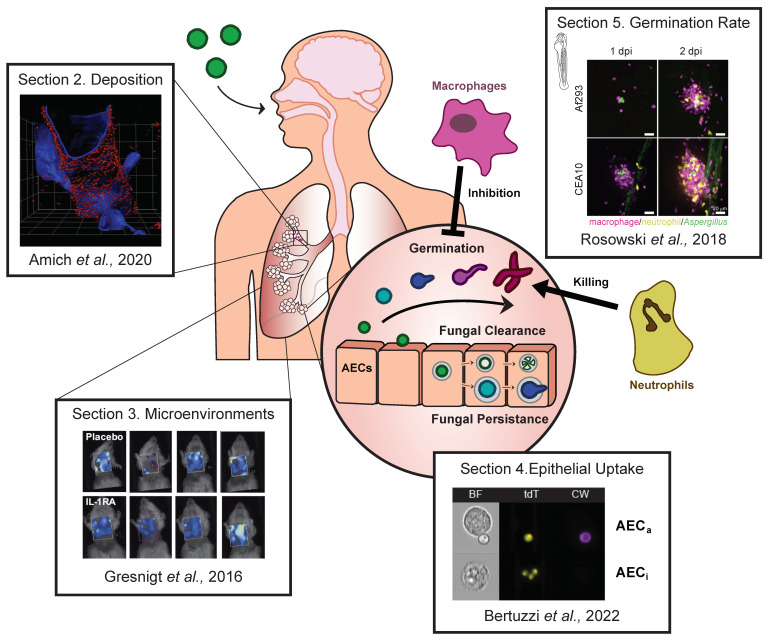
Diagram of new insights into *A. fumigatus* host–pathogen interactions attained through novel in vivo imaging techniques. Section 2 : Work by Amich et al. (2020) used light sheet fluorescence microscopy (LSFM) to look at *A. fumigatus* lung deposition [15]. Section 3: Gresnigt et al. (2016) investigated the interplay between hypoxic lung environments and inflammation during *A. fumigatus* infection using fluorescence tomography [16]. Section 4: Bertuzzi et al. (2022) leveraged imaging flow cytometry (IFC) to demonstrate in vivo uptake and killing of *A. fumigatus* by airway epithelial cells (AECs) (BF: bright field, tdT: tdTomato identifying interacting *A. fumigatus*, CW: calcofluor white identifying external *A. fumigatus*) [17]. Section 5: Rosowski et al. (2018) used a zebrafish model of aspergillosis to demonstrate that germination rate can alter neu-trophil recruitment and antifungal activity while also being modulated by macrophages [18].

Despite staggering morbidity and mortality, the mechanisms underlying *A. fumigatus* pathogenesis and the concomitant host response are not fully elucidated. This has resulted in a limited availability of effective antifungal and immunomodulatory therapies to combat *Aspergillus*-related diseases. We aim to review the advanced imaging techniques applied to invertebrate and murine models of aspergillosis, which have recently provided important visually-quantitative insights into the host–pathogen interactions within the infected host. Previous reviews have already expertly addressed the current knowledge on, and dynamic intricacy of, the *A. fumigatus* host–pathogen interaction (some examples are [10,11,12,13,14]); therefore, in this review, we will discuss recent contributions and future potential applications of state-of-the-art imaging techniques by answering four outstanding questions regarding *A. fumigatus* pathogenesis and antifungal response during *A. fumigatus* infection (Figure 1) [15,16,17,18].

## 2. Are *A. fumigatus* Spores Deposited in the Alveoli?

It is estimated that humans breathe in between 1000 and 10 billion fungal spores a day [19]. These spores are routinely cleared in healthy individuals through a variety of defence mechanisms, including mucociliary clearance and the phagocytic and antimicrobial activities of resident epithelial cells and innate immune cells [20,21]; however, in certain individuals, *A. fumigatus* conidia bypass these defences, leading to disease. *A. fumigatus* conidial concentrations in the environment are estimated to be between 1 and 100 conidia/m^3^, with the average adult likely inhaling more than 100 conidia daily [1]. Due to the relatively small size of *A. fumigatus* conidia (2–3.5 µm), the field has largely assumed that these spores are able to reach the small airways and the alveoli, a feature critical to their pathogenesis, making the alveoli the principal site of invasive pulmonary aspergillosis (IPA) [1,22,23,24,25]. Lung deposition likely dictates the cellular milieu faced by inhaled *A. fumigatus* spores, which is also likely to differ across clinical scenarios with predispositions for infection. Consequently, gaining insights into the initial encounters of *A. fumigatus* within the complex spatial microarchitecture of respiratory airways has significant implications for the design of targeted therapeutic interventions. Unfortunately, technical and ethical constraints have led to difficulties in assessing the extent of alveolar deposition in mammalian diseased lungs. To this end, recent technical advancements in whole organ imaging have enabled the characterisation of *A. fumigatus* conidial lung deposition in murine models of infection [15].

The development of whole lung clearing and three-dimensional light sheet fluorescence microscopy (LSFM) have resulted in the ability to image entire lung lobes at cellular resolution, thereby enabling, in combination with fluorescent staining, the characterisation of fungal growth and host immune recruitment within the spatial-anatomical context of an infected lung [15]. Using a previously established lung clearing protocol to image deep into the host lung [26], fluorescently labelled antibodies to identify host immune populations and JF5 (an *Aspergillus*-specific monoclonal antibody which binds to an *Aspergillus* mannoprotein antigen secreted during active growth) to visualise growing *A. fumigatus* [27], LSFM was employed to establish *A. fumigatus* growth under standard immunosuppressive murine models of IPA [15]. Using this technique, Amich et al. (2020) quantified fungal burden in a highly sensitive manner and characterised key immune populations to reveal changes to pulmonary lung environments upon *A. fumigatus* infection. Importantly, this study was also able to investigate *A. fumigatus* deposition across murine lungs following intranasal infections. Epithelial surfaces were visualised using a fluorescently labelled anti-podoplanin antibody, and location of fungal burden was denoted based on the typical dimension of murine alveoli [15,28,29]. After 16 h of infection, only ~20% of actively growing cells co-localised in the alveoli, with ~40% co-localising with bronchioles and 20% in close proximity of the bronchioles (see Figure 1). These results suggest that during intranasal infections with conidia, in standard murine models of IPA, the largest burden of actively growing *A. fumigatus* after 16 h of infection is not in the alveoli, but rather in the bronchioles. Furthermore, minimal alveolar deposition was observed irrespective of the immunosuppressive model of pulmonary aspergillosis adopted, based on immunosuppression with corticosteroids, chemotherapy-induced leukopenia or myeloablative irradiation.

While these results are extremely valuable to our current interpretation of murine models of IPA, there are caveats to expanding this to our understanding of *A. fumigatus* conidial deposition in humans, as highlighted by the authors as well. First of all, there are morphological differences between the murine and human respiratory tracts, which are likely to affect the ability of small particles to be deposited in this region. Specifically, murine alveoli are significantly smaller than human ones, with the alveolar surface and the alveolar diameter being, respectively, 21× and 4× smaller in mice than in humans [30]. Moreover, the use of the JF5 antibody limits the imaging and quantification exclusively to actively growing *A. fumigatus.* This antibody does in fact only visualise hyphal cells because it selectively binds to an *Aspergillus* mannoprotein antigen secreted during active growth [27]. In addition, due to the time point selected for the assessment of fungal burden (16 h), there is no accounting for early anti-fungal clearance by epithelial and immune cells in the alveoli. This is a crucial point since internalisation of conidia by epithelial and immune cells and the derived immune responses are also known to occur within the first 16 h of contact [31,32]. Since only actively growing *A. fumigatus* cells are being labelled and quantified, any conidia whose germination and growth has been already inhibited or delayed might be excluded from this experimental setup. Therefore, it is possible that a larger proportion of conidia reaches the alveolar region than reported, but that the effective fungicidal and fungistatic activities occurring over the first 16 h leads to an underestimation of the fungal deposition observed in the alveolar region. Finally, aerosolised conidia likely deposit differently than conidia in a liquid suspension. While inhalation of fungal spores by humans generally occurs with aerosolised spores, standard murine models entail the resuspension of spores in water or, as in this case, in water + 0.9% NaCl + 0.005% Tween 20 for intranasal instillation. However, this difference in fluidics likely alters the deposition of conidia and does not necessarily reflect the natural progression of infection. Taken together, these could explain why the results from whole-lung LSFM conflict with the general assumption that *A. fumigatus* conidia largely deposit in the small airways and alveoli. 

Indeed, Geiser et al. (2000) measured the distribution of aerosolised *Calvatia excipuliformis* (puffball) basidiospores across the lungs of hamsters immediately after inhalation (fixed within 29 min), demonstrating that fungal spores of ~3 µm in size largely deposit in alveoli [33]. Using a combination of lung sectioning and histology, it was found that two-thirds of spores were in the alveoli both when hamsters inhaled the aerosolised spores by spontaneous breathing or by continuous negative-pressure ventilation. Importantly, these spores are similar in size to *A. fumigatus* conidia with an aerodynamic diameter of 3 µm but factors other than size, such as hydrophobicity and electrical charge, could alter the deposition of spores, thus a direct comparison of *C. excipuliformis* to *A. fumigatus* cannot be made. However, while using less complex techniques, this study overcomes many of the caveats described above, such as using aerosolised spores for infection, quantifying burden immediately and, finally, using hamsters, which have slightly larger alveolar regions than mice [30]. Taken together, these experimental differences may explain the significant variations in results between the two studies. Similarly, histology-based investigation of *A. fumigatus* has primarily identified alveolar deposition [34]. Numerous sampling studies of environmental spores have also established models of lung deposition that support the idea of alveolar *A. fumigatus* conidial deposition. For instance, deposition models following spore sampling in residences in the Northeastern U.S. calculated the fractional deposition for the *Aspergillus* genera in the extrathoracic (ET; 66%), tracheobronchial (TB; 7%) and alveolar (AL; 27%) regions of the human respiratory tract based on local measurements of spore concentrations and the physiological data of residents [35]. While the majority of spores are deposited in the ET region, *Aspergillus* was one of the top occupiers in the alveolar region compared to other environmental genera, which is consistent with other *Aspergillus* deposition models [36,37,38,39]. 

While not directly addressing whether *A. fumigatus* conidia are primarily deposited in the alveoli of humans, the approach presented by Amich et al. (2020) provides the first-in-field experimental data characterising *A. fumigatus* conidia deposition within the context of murine lung architecture and provides significant insights into our current murine IPA models. LSFM is a non-destructive method that avoids the need to process samples into single-cell suspensions or slices as well as the risk of inaccurately representing the cellular composition of the sample itself. This is in comparison to standard techniques, such as colony-forming units (CFU) enumeration or qPCR, all of which requiring lung homogenisation. LSFM is also sensitive enough to quantify fungal burden and immune cells deep in the organ and can enumerate ~5× more cells than flow cytometry while maintaining the spatial context [15]. The holistic spatial information attained allows for characterisation of the immune cell associations around specific *A. fumigatus* clusters distributed throughout the lung, which is information often lost in other techniques that require sample processing. Prior to LSFM development, the main option for obtaining insights on fungal burden and immune cell distribution within an intact anatomical context had been histology. However, histology is a time- and labour-intensive process and is often practically unfeasible for whole lungs, as it relies on thin consecutive lung sectioning followed by staining, thus reducing imaging capabilities to only small areas and possibly resulting in sampling bias. High-throughput slide scanning microscopes are beginning to allow the acquisition and assembly of entire histological sections in a faster manner but will still lack the ability to return the holistic 3D architecture of the infected organ, as achieved by LSFM. Because of these constrains, histological data have often served as a useful reference for the assessment of fungal germination and growth, as well as for the presence of immune cells, but are often limited to qualitative data, whereas LSFM provides a novel method of quantitative assessment. 

The most notable limitations of the LSFM technology include laborious and expensive sample preparation, whereby staining and clearing procedures can take several days and large amounts of antibodies are often required. LSFM also requires a specialised light sheet microscope with expertise to acquire and analyse the complex 3D data. Moreover, tissue fixation is necessary, and, therefore, the analyses are limited to acquisition of the single time points of the infection process. While able to rapidly image whole organs, LSFM has a lower spatial resolution than confocal laser-scanning microscopy, which has been recently used by Bogorodskiy et al. (2020) to look at the behaviour of intra-epithelial dendritic cells during *A. fumigatus* infections [40,41]. This methodology uses a similar lung clearing procedure to that in Amich et al. (2020) but coarsely sections the lung into thick slices to visualise the host–pathogen interactions. Interestingly, via tracking deposition using dTomato-expressing conidia, rather than hyphae-labelling with the JF5 antibody, this study found conidial accumulation in the alveolar space, with less presence in the bronchial region after 72 h of infection. 

LSFM provides a novel method of investigating previously uncharacterised aspects of *A. fumigatus* pathogenesis. For example, the use of aerosolised spores, rather than spores resuspended in liquid, and the quantification of conidial burden using a non-hyphal morphotype-specific antibody immediately after infection, might lead to a better picture of the natural deposition of *A. fumigatus* conidia. Additionally, these experiments could be attempted in larger animal models that have larger (more human-like) lung structures. LSFM could also be used to characterise changes in the levels of deposition in different models of disease to elucidate key host factors and the host–pathogen interactions influencing disease kinetics in at-risk patients. For instance, given that COPD and CF are known to significantly affect lung architecture and are known to be key risk factors for the development of IPA and ABPA, respectively [5,42,43,44], characterising the variations in deposition across the lungs in specific murine models of these diseases could reveal new insights surrounding these risk factors [45]. The ability of *A. fumigatus* to efficiently colonise and infect humans has been postulated to be, in part, due to its conidial size (2–3.5 µm), which is smaller than those of other pathogens like *A. flavus* and *A. niger* (3–5 µm), hence favouring avoidance of mucociliary clearance and deeper lung deposition [25,46]. Thus, characterising the changes in fungal growth and in immune recruitment in these models of disease could elucidate key host factors and the host–pathogen interactions influencing disease kinetics. Additionally, LSFM could be used to compare levels of deposition between pathogenic and non-pathogenic *Aspergillus* strains and species to establish if there is indeed a correlation between spore size, lung deposition and pathogenicity. Finally, by performing LSFM at multiple early time points of infection, the specific immune cell populations being recruited during infection could be identified. In all, while LSFM has not yet fully established whether *A. fumigatus* conidia are deposited in the human alveoli, it does represent a powerful and innovative technique that may enable us to develop a better understanding of the early events in *A. fumigatus* infection and disease.

## 3. What Microenvironments Does *A. fumigatus* Encounter upon Lung Deposition?

Once deposited in the lung, spores encounter complex microenvironments, whose characteristics (for example: oxygen composition, pH, metals and nutrient availability) can impact fungal stress adaption, conidial germination and hyphal growth, uptake by host cells as well as antifungal defence, thereby ultimately altering disease kinetics. The ability to detect and measure local tissue parameters in vivo is crucial, and key advances in state-of-the-art imaging of host–pathogen interaction can help to characterise the role that these microenvironments play in the interactions of *A. fumigatus* with the host and, thus, elucidate their relevance to disease to develop innovative therapeutic interventions. A key example of this has been in the exploration of the role of hypoxic lung microenvironments during IPA establishment, which has directly led to the identification of novel potential therapeutic avenues [16,47]. Oxygen levels are a key, yet highly heterogenous, component of the lung environment [48,49]. The levels of oxygen across the lung are lower than atmospheric concentrations (~21% O_2_), and the highly oxygenated alveoli, which are the putative main site of initial infection during IPA (see Section 2), contain ~14% O_2_ in lungs of healthy individuals. However, the actual oxygen availability is likely to be lower due to haemoglobin’s high affinity for O_2_ [48,49]. Additionally, infected wounds and abscesses have lower O_2_ levels than their surrounding tissues, suggesting lower oxygen levels at infection sites, such as aspergillomas [48,50,51]. 

A study by Grahl et al. (2011) was the first to identify the hypoxic microenvironments associated with *A. fumigatus* in murine models [47]. This study investigated metabolite production during IPA by performing nuclear magnetic resonance (NMR) analyses on bronchioalveolar lavages (BAL) from infected mice and identified ethanol as an overrepresented metabolite during disease. *A. fumigatus* produces ethanol under hypoxia, thereby leading to the hypothesis that *A. fumigatus* encounters hypoxic conditions during IPA. To test this hypothesis and visualise hypoxic environments in vivo, this study used the nitroheterocyclic compound Hypoxyprobe^TM^ (pimonidazole hydrochloride), whose activation is hypoxia-dependent and leads to the production of protein adducts that can be visualised using immunofluorescence [47,52]. Using a combination of Hypoxyprobe^TM^, sectioning and immunohistochemistry, in vivo hypoxia was investigated in three immunosuppressive models (chemotherapeutic, corticosteroid and X-CGD). Across all three models, variable levels of inflammation, fungal growth and hypoxia were detected; however, every model showed clear hypoxic microenvironments 3–5 days post infection. Variation across the immunosuppressive models suggested that host inflammation in these different models could play a role in generating these hypoxic microenvironments. Though a few previous studies indicated a potential role of hypoxia in *A. fumigatus* pathogenesis, this study was the first demonstration of hypoxic microenvironments during *A. fumigatus* infections and initiated further studies into the role of hypoxia on *A. fumigatus* virulence and pathogenesis, as well as its potential exploitation as a novel target for therapeutic intervention [47,53,54,55,56,57].

Numerous studies have explored the interplay between hypoxia and aspergillosis, linking the ability to grow in hypoxic conditions to pathogenesis [54,58,59,60], identifying the genes essential for both hypoxia adaptation and virulence [56,61,62,63,64,65] and characterising hypoxia factors through transcriptomics [66,67,68], proteomics [66,69,70,71] and metabolite identification [69,72]. The effect of hypoxic conditions on antifungal efficiency, drug resistance and diagnostic efficacy has also been investigated [48,73,74,75]. As such, the potential role of hypoxia in aspergillosis has been discussed in detail throughout multiple reviews [53,76,77,78,79]. Along with the many studies exploring the role of hypoxia in *A. fumigatus* biology and disease, a recent notable study by Gresnigt et al. (2016) leveraged fluorescence tomography to investigate the interplay between inflammation, hypoxia and fungal growth in murine models of IPA [16]. This study used fluorescence tomography and an HS680 probe (HypoxiSense 680), previously used to measure tumour hypoxia [80], to identify and quantify hypoxic lung microenvironments in a corticosteroid IPA model (see Figure 1). In parallel, bioluminescence imaging was also performed to gain a temporal assessment of fungal burden using luciferase-expressing *A. fumigatus* strains [55]. Corticosteroid-immunosuppressed mice showed higher levels of hypoxia (as determined by the HS680 signal) and higher fungal burdens (as determined by luminescence) than immunocompetent mice. Importantly, this in turn correlated with significantly higher inflammation as determined by pro-inflammatory cytokine levels (CXCL1, IL-1α, IL-1β, IL-6 and G-CSF) in lung homogenate 3 days post infection (dpi). 

Host inflammatory responses during aspergillosis must balance the clearance of *A. fumigatus* and limitation of tissue damage [81], and hypoxia is a key player in this balance since it can increase inflammation, which in turn leads to acute lung injury, in a vicious cycle where more tissue damage can lead to further tissue hypoxia. The interplay between hypoxia and inflammation therefore represents a potential therapeutic target in lung disease [82]. A key master transcriptional regulator for inflammation under hypoxic conditions is the hypoxia-inducible factor-1 alpha (HIF-1α) which is known to regulate Interleukin-1 alpha (IL-1α) [83], a crucial component of the host defences against *A. fumigatus*. IL-1α mis-regulation can, however, lead to inflammatory disease [84,85] and, on the basis of this, Gresnigt et al. (2016) hypothesised that inhibition of IL-1 signalling via an IL-1 receptor antagonist (IL-1Ra, Anakinra) could disrupt this vicious cycle of hypoxia-inflammation during IPA and potentially minimise disease. Accordingly, when *A. fumigatus*-infected and immunosuppressed mice were treated with IL-1Ra, there was a significant decrease in hypoxia compared to untreated mice, and a sub-population of the treated mice (6/10) showed significantly reduced pulmonary inflammation. Importantly, when mice were infected with a low dose of conidia (2 × 10^5^), IL-1Ra treatment led to a significant decrease in fungal burden at 2 dpi (as determined by bioluminescent signal) and an improvement in wellbeing at 3 dpi (as determined by body weight). Taken together, this study used live imaging to investigate the connection between IL-1α-mediated inflammation and hypoxic microenvironments during IPA in mice, with the aim of exploring this axis for therapeutic intervention. Indeed, the results presented suggest that targeting immune signalling (like that of IL-1α) could help reduce hypoxia and improve the outcomes of IPA; however, whether targeting host factors to modulate inflammation and hypoxia is a viable method of treatment for IPA in humans is still to be determined. Hypoxia has been shown to modulate immune responses against *A. fumigatus*, both positively (by inducing fungal cell wall changes and enhancing effector cell activation) and negatively (through attenuation of human dendritic cell activation) [86,87]. Minimising hypoxia for therapeutic purposes could also be used in combination with current antifungal treatments. Gresnigt et al. (2016) showed preliminary evidence that a combination treatment of IL-1Ra (10 mg/kg/day) with caspofungin (10 mg/kg/day) was well tolerated by mice and improved their survival from 40% to 60% in the case of treatment with caspofungin alone [16]. Implementing hypoxia/inflammation modulation with an antifungal treatment could be particularly fruitful from a clinical point of view, given that, even though hypoxic conditions only minimally alter antifungal effectiveness against aspergilli in vitro, hypoxic microenvironments have been demonstrated to drive antifungal resistance in *A. fumigatus* [48,73,74].

The implementation of fluorescence tomography to explore the complex lung microenvironments in the context of aspergillosis is promising. There are many major benefits of fluorescence tomography, including that it can be used for longitudinal studies, whereby it provides a method for temporal assessment of parameters in the same mice over time, thus significantly reducing the number of mice needed for infections. It is important to note, however, that the application of this technology, together with the quantification of fungal burden in *A. fumigatus* via luminescence strains, can be hampered due to the intrinsic requirement of oxygen for enzymatic-dependent detection of these strains [55,57,88,89,90,91,92]. This is particularly important considering our current understanding of hypoxic microenvironments during *A. fumigatus* infections and has been indeed acknowledged by the authors of multiple studies [16,47,53,55]. Further non-invasive imaging techniques of live infected mice include combining bioluminescence with magnetic resonance imaging (MRI) to both obtain dynamic information of fungal burden (from bioluminescence readout) and lesion number and size (from MRI readout) in a non-invasive manner [93]. MRI information is particularly beneficial for late-stage disease where oxygen limitation due to inflammation can cause issues in luminescence resolution [93]. Another avenue has been the development of the hyphal-specific humanised monoclonal antibody hJF5, which has been radiolabelled ([^64^Cu]DOTA-JF5) as a tool for the monitoring of pulmonary aspergillosis using antibody-guided positron emission tomography and magnetic resonance (immunoPET/MRI) [27]. This technique has shown promise for clinical diagnostics purposes, rather than as a tool to investigate pathogenesis mechanisms in murine models of aspergillosis [94,95]. Further engineering of the hJF5 antibody has facilitated more exciting combinations of imaging using a dual-labelled hJF5 with both radionuclide and fluorophore (^64^Cu-hJF5-DyLight650) for its use in both immunoPET/MRI, which enables broad resolution longitudinal studies in live mice and LSFM of optically cleared dissected lungs, thereby allowing for higher-resolution imaging [96]. The technical hurdles and costs of immunoPET/MRI can still be prohibitive, but this technique could facilitate unique in vivo studies.

Following the crucial discovery of hypoxic microenvironments during IPA [47], fluorescence tomography has allowed for in-depth characterisation of the interplay between hypoxia and inflammation, as well as of its potential use as a target for therapeutic intervention against aspergillosis [16]. Fluorescence tomography and other in vivo imaging techniques could be further exploited to study the contribution of fungal and host components in the establishment of hypoxic microenvironments in infected lungs. For example, it has previously been shown that *A. fumigatus* strains that thrive in low oxygen conditions are often more virulent [59]; therefore, the evaluation of hypoxia levels during infection with different low oxygen-growing strains could lead to a better understanding of the factors of *A. fumigatus* that may drive hypoxic environments or modulate the host response to alter oxygen levels. Although hypoxia is a clinical feature of both COPD and CF lungs [97,98], links between the hypoxic microenvironments in the lung and the susceptibility of COPD and CF sufferers to aspergillosis have yet to be investigated. Using CF murine models, hypoxia could be evaluated in the context of aspergillosis [45,99]. Fluorescence tomography could also be further developed to identify other microenvironments that alter host–pathogen interactions and disease kinetics. For instance, both pH and zinc have been implicated in the virulence of *A. fumigatus* [31,100,101,102,103], and fluorescence tomography could be used to evaluate pH variation and zinc levels during IPA using in vivo probes such as LS482 for pH and ZPP2 for zinc [104,105]. Determining the local pH and zinc landscape during IPA could open the doors to novel avenues of research and direct us towards unexplored therapeutic targets. As more probes are developed for in vivo imaging, other factors such as temperature, oxidative stress, metals and nutrient availability could be probed. As research emerges implicating specific environmental factors in *A. fumigatus* pathogenesis, in vivo imaging techniques provide exciting modular methods to characterise multiple factors in the context of host–pathogen interactions and disease. 

## 4. Are Airway Respiratory Cells Internalising *A. fumigatus* during Infection?

Following inhalation and deposition in the respiratory tract, healthy clearance of *A. fumigatus* conidia has largely been attributed to the concerted action of resident and recruited innate immune cells, such as alveolar macrophages (AM) and neutrophils [106,107,108,109,110]. However, murine studies [110] and in silico modelling [111,112] indicate that AMs migrate too slowly and randomly in the absence of a chemoattractant signal to be able to protect against *A. fumigatus* infection and suggest that neutrophils are recruited at a later stage to the site of infection and are, therefore, unlikely to play a key role in immediate spore clearance [110,111,113,114]. Additionally, AMs only constitute ~3% of the total cell number in the alveoli [115] and, therefore, are unlikely to be the first cell type encountered by the inhaled spores. Given this evidence, it is likely that other resident lung cells could play important roles in the initial host–pathogen interactions leading to healthy clearance of *A. fumigatus*. For instance, bronchial and alveolar epithelial cells, collectively referred to here as airway epithelial cells (AECs), cover the entire alveolar surface and comprise 24% of all cells in human lung parenchyma [115]; therefore, their contact with *A. fumigatus* conidia is instant, extensive and likely prolonged. AECs are found to play a crucial role in host defence against various respiratory pathogens by orchestrating highly multifaceted responses and promoting the uptake and killing of inhaled microbes [116]. Recent in silico modelling that incorporated the role of AECs in the host response to *A. fumigatus* suggests that AECs play a key role in fungal clearance and may be the primary cell type releasing cytokines for immune cell recruitment during infection, rather than AMs [117,118,119]. The role of AECs in host defence against *A. fumigatus* infections is just starting to be explored, but a variety of recent evidence suggests that these non-professional phagocytic cells likely play a key role in fungal clearance and host response (as reviewed by [21,32,119]).

Numerous studies have demonstrated that *A. fumigatus* conidia are readily internalised by AECs in vitro, with both alveolar and bronchial epithelial cells internalising 30–50% of the conidia they encounter [17,31,120,121,122,123,124,125,126,127,128,129]. In vitro *A. fumigatus* uptake by AECs is a time-dependent process, which increases proportionally with longer co-incubations or a higher multiplicity of infections [17,126,128]. AECs exert antifungal activity, with intracellular conidia showing impaired germination relative to extracellular conidia [17,126]. This antifungal activity has been largely attributed to phagosome acidification [122,126], and, indeed, population- and single-cell studies have demonstrated that the vast majority of internalised conidia (90–97%) are killed by AECs within 12–24 h of infection; however, a small subpopulation is able to avoid clearance and to germinate intracellularly [17,122]. In response to challenging intracellular conditions, this subpopulation shows unique morphological changes and surviving intracellular conidia frequently form secondary germ tubes and contain an increased number of vacuoles in their hyphae [126]. Interestingly, AECs that internalise conidia do not undergo increased cell death (relative to uninfected cells), suggesting that, while *A. fumigatus* clearly leads to damage to AEC monolayers, this damage is not primarily associated with uptake [17]. Indeed, a small proportion of germinated intracellular conidia are able to escape AECs while avoiding the rupturing of host cells, and, following non-lytic escape, hyphal extensions can invade the adjacent AECs without further perpetuating host cell damage [126,130]. Importantly, hyphae escaping from AECs are coated in host plasma membrane, and this might enable *A. fumigatus* to evade the host immune cells [126]. In addition to uptake, AECs have been shown to respond to the challenge of *A. fumigatus* with an organised and regulated host response, involving the synergistic expression and release of several immune mediators [117,131,132,133,134,135,136,137,138]. Taken together, this evidence indicates that AECs play an important role in efficient fungal clearance and host response following everyday exposure to *A. fumigatus* conidia. However, despite a variety of pathogen and host factors identified as playing roles in *A. fumigatus* uptake by AECs [21,32,139,140], the functional relevance of this process to disease outcome remains unclear, and novel techniques are needed to determine whether AECs internalise *A. fumigatus* during mammalian infection. 

Initial experiments in murine models used transmission electron microscopy (TEM) on histopathological sections to quantify the conidial uptake by bronchial cells in corticosteroid models of infection [34]. These experiments were unable to identify any internalisation events by the bronchial epithelial cells at 6 and 18 h post infection. However, due to the poor sensitivity of TEM and the low frequency of uptake by AECs in vitro [17,141], in vivo internalisation by the bronchial epithelial cells cannot be excluded. Additionally, this study focused solely on identifying in vivo uptake by bronchial epithelial cells, and uptake by alveolar epithelial cells was not explored. Indeed, Bertuzzi et al. (2022) recently coupled differential fluorescence staining and imaging flow cytometry (IFC) to demonstrate and quantify in vivo uptake of *A. fumigatus* by AECs in a leukopenic murine model of IPA [17,141]. AECs were isolated from dissociated lungs of the leukopenic mice infected for 8 h with tdTomato-expressing *A. fumigatus* strains. Prior to IFC analysis, staining of *A. fumigatus*—AEC complexes with the cell impermeant dye calcofluor white allowed for differentiation of the AECs that had internalised *A. fumigatus* or had *A. fumigatus* attached to their surface (see Figure 1), while staining with antibodies against EPCAM, podoplanin and CD74 allowed for typing of murine bronchial, type-I and type-II alveolar epithelial cells [17]. Using this optimised experimental pipeline, it was found that 3.5% of murine type-II alveolar epithelial cells internalise *A. fumigatus*. Importantly, this provided the first ever in vivo evidence of *A. fumigatus* uptake by AECs, demonstrating that primary murine type-II epithelial cells internalise *A. fumigatus* at rate and stoichiometry similar to cultured type-II epithelial cells [17]. Furthermore, by fluorescence-activated cell sorting (FACS), this study also assessed the ability of AECs to kill internalised conidia in vivo. Type-II AECs were able to kill 69.9% and >97% of the internalised fungi following 4 and 16 h of infection respectively, in strong agreement with previous in vitro evidence [17,122,141]. 

Given that AECs provide a potent means of antifungal defence against *A. fumigatus* in vivo, dysfunctional epithelial protective activities in at-risk patients may provide an opportunity for *A. fumigatus* to exploit AECs as a safe haven in which to reside intracellularly, thereby escaping immune cells’ recognition and clearance. A variety of pre-existing conditions are known as key risk factors for aspergillosis (such as COPD, asthma and CF) [2,3,4,5,6]. Indeed, a study by Chaudhary et al. (2012) showed that the uptake and killing of conidia by bronchial epithelial cells is impaired by the presence of a non-functional cystic fibrosis transmembrane conductance regulator (CFTR, DF508), thereby supporting the view that dysfunctional epithelial responses might at least contribute, if not be the basis of, increased susceptibility to aspergillosis in at-risk patients [142]. Following this hypothesis and considering that recent estimates indicate that nearly 3.9% of COPD patients admitted to hospital annually develop IA, with 540,451–977,082 predicted deaths annually [5], Bertuzzi et al. (2022) went on to test the ability of AECs derived from patients with COPD to internalise and kill *A. fumigatus.* The COPD-derived primary human AECs were found to internalise conidia more efficiently than their healthy counterparts (~4 and ~2 times for commercially available and locally-sourced AECs, respectively); however, importantly, gorging COPD-derived AECs were also half as capable of killing internalised conidia [17]. The observed dysregulation of epithelial protective activities in primary human diseased AECs [17,142] provides a novel understanding of how the disease kinetics of *Aspergillus* infections are altered in at-risk patients, suggesting that AECs play a key role in antifungal defence at very early stages of the infection process and are, therefore, an appealing target for therapeutic intervention. 

This innovative single-cell approach allowed for the first ever evidence and quantification of in vivo *A. fumigatus* uptake by AECs and demonstrated that uptake results in effective killing of the internalised fungi, not only in vitro, but, importantly, also during mammalian infection. A drawback of this technique is that the analysed cells have to be in suspension; therefore, when starting from in vitro infections, the infected epithelial monolayers need to be disaggregated and detached, whereas when starting from in vivo infections, the infected organs need to be extracted and dissociated. Thus, unlike LSFM (see Section 2), IFC does not place the host–pathogen interactions in the context of the 3D architecture of an intact lung, and, unlike fluorescence tomography (see Section 3), IFC cannot provide longitudinal data on infection burdens and lung environments in live mice. However, overshadowing this drawback, IFC offers the unique and crucial advantage of enabling assessment of the host–pathogen interactions in vitro or in vivo with single-cell resolution, even when these interactions are relatively rare events. The combination of IFC with different fluorescent stains and dyes provides a flexible toolkit for an in-depth analysis of the host–pathogen interactions, which permits simultaneous testing of distinct host cell populations (in addition to AECs) interacting with different morphotypes or mutants of *A. fumigatus* in vitro or in vivo. Importantly, this could lead to the identification of pathogen factors that could be inhibited or host immunomodulatory targets that could be potentiated for therapeutic purposes. 

## 5. How Does Germination Alter the Interactions of *A. fumigatus* with Immune Cells?

The genetic programming driving the germination of fungal spores into vegetatively growing cells is poorly understood, yet the process of germination is essential for the majority of spore-mediated fungal diseases [143,144,145]. For *A. fumigatus,* the process of germination involves a multi-step morphological transition, whereby small, circular, resting conidia undergo isotropic growth into large, swollen conidia, which form germ tubes and later progress into hyphae. Throughout these morphological changes, epitopes on the surface of *A. fumigatus* change significantly, with important implications for immune recognition by the host [21,146,147,148]. Accordingly, different morphotypes are known to interact differently with host immune cells and elicit varied immune responses [21,134,146,148,149,150,151,152,153,154]. In turn, the host cells inhibit germination to varying degrees. For example, in vitro evidence suggests that the antifungal activity of neutrophils is primarily targeted at the hyphal morphotype rather than conidia [155,156]; however, evaluation of the fungal and host composition of the bronchioalveolar lavage fluid (BALF) from infected mice suggests that neutrophil aggregates inhibit the germination of *A. fumigatus* conidia in an ROS-dependent manner [113]. Macrophages can inhibit the germination of intracellular conidia in vitro via ROS-dependent and independent mechanisms [157,158,159], and this inhibition is crucial to fungal clearance, as intracellular germination of *A. fumigatus* otherwise drives programmed macrophage necrosis in vitro [154]. Finally, in vitro AECs inhibit the germination of both internalised and external conidia, but internalised conidia are more efficiently killed as a result of phagolysosomal trafficking and acidification [17,120,122,126,129,160,161,162]. Taken together, this evidence suggests that the process of germination in vivo might dictate variations in morphotype-dependent interactions with the host and, in turn, the disease kinetics of *A. fumigatus*. 

While germination is clearly a key driver of host–pathogen interactions, its role in shaping the disease kinetics of *A. fumigatus* is just starting to be investigated. A comparison of *A. fumigatus*, *A. nidulans*, *A. niger* and *A. terreus* shows that faster germination rates correlate with a higher cytotoxicity than those of cultured AECs in vitro (A549 cells) [117,163]. Similarly, when infecting macrophages in vitro with *A. nidulans* and *A. fumigatus*, the former germinates more readily than the latter, but differences in uptake, phagosome acidification, fungal killing, phagocyte migration and cytokine induction are also observed, thereby preventing a clear indication of the role of germination in the species-specific host–pathogen interaction [164]. Importantly, while *A. nidulans* germinates the fastest and causes the most damage in vitro, it is not a leading cause of IA [165], which suggests that germination rates in vitro do not always directly mirror the disease kinetics in the host. Along these lines, an evaluation of five *A. fumigatus* strains with different germination rates across different models of infection demonstrated that there was a large variation in virulence across strains, depending on the model used, but no direct correlation to their germination rate [166]. To better understand the role of germination in aspergillosis, two commonly used *A. fumigatus* clinical isolates, namely CEA10 and Af293, have been thoroughly investigated in murine and zebrafish models of IA [31,59,166,167,168]. Through histological analysis and evaluation of the BALF of infected murine lungs, Caffrey-Carr et al. (2017) found that CEA10 germinates faster than Af293 in vivo, resulting in increased lung damage, inflammation due to IL-1α-dependent neutrophil recruitment and increased virulence in IL-1α^−/−^ mice. Importantly, infection of macrophage cultures with Af293 germlings was sufficient to induce equivalent damage and IL-1α release as with CEA10 germlings, which suggests that this was a morphotype-specific host response, absent with conidia. Germlings needed to be alive for IL-1α induction, suggesting that the surface-exposed factors of hyphae were insufficient for this response and that morphotype-dependent secreted factors from *A. fumigatus* may modulate the host response dramatically [138,167]. Visualising where and when these secreted factors act, using similar fluorescent protein technology to that applied in the study of bacterial effector translocation [169], will therefore be a key step to understanding the impact of fungal germination and development on *A. fumigatus* disease kinetics. 

These results indicate that germination upon lung deposition is a key factor in determining disease outcomes, yet detailed characterisation of the germination of individual fungal cells is not technically feasible in murine models. This is due to the lack of high-resolution imaging technology to assess fungal morphotypes in live mice using longitudinal studies. Crucially, the recent development of an optically-clear zebrafish model of IA by Knox et al. (2014) has permitted the use of 4D confocal or widefield microscopy to visually quantify fungal development, immune cell recruitment and the *A. fumigatus*–host interactions in vivo [170,171,172,173,174]. Using this model, Rosowski et al. (2018) were able to demonstrate that variations in germination rates among *A. fumigatus* strains could shape disease kinetics [18]. As mentioned previously, CEA10 germinates faster than Af293, both in vitro and in murine and zebrafish models [167,168]; however, in vivo germination of individual *A. fumigatus* cells had never been monitored before. Thanks to the possibility to image infected zebrafish daily, Rosowski et al. (2018) directly quantified fungal germination and growth in larvae for 5 days [18]. Indeed, the CEA10 isolate was found to germinate and grow into mature hyphae faster than the Af293 isolate, with 20% more larvae infected with CEA10 containing the germinated *A. fumigatus* than those infected with Af293 (in immunocompetent larvae) as early as 2 days post infection. On the other hand, the authors found that Af293 was less efficiently killed than CEA10 in immunocompetent fish, which was surprising given that, while neither strain is virulent in immunocompetent fish, CEA10 is more virulent in neutrophil-deficient zebrafish [168]. In zebrafish, neutrophils are known to preferentially migrate and associate with hyphal forms of *A. fumigatus*; therefore, to understand the preferential killing of CEA10, Rosowski et al. (2018) were able leverage the in vivo cellular imaging of larval zebrafish to link the germination rate with increased neutrophil killing activity [18,170].

The ability to visualise specific immune cell clusters over time in individual zebrafish provides a temporal assessment of recruitment of specific populations for distinct infection events. By exploiting zebrafish with fluorescently labelled phagocytes, Rosowski et al. (2018) were able to monitor variations in macrophage and neutrophil recruitment and behaviour in individual larvae over 5 days of infections. Compared with Af293 infections, the CEA10 strain was found to elicit over two-fold greater recruitment of both macrophages and neutrophils as early as 2 dpi and correlated with significantly larger immune cell clusters surrounding *A. fumigatus* (see Figure 1). Recruited phagocytes, initially mainly macrophages, formed tight clusters around *A. fumigatus* as early as 1 dpi and lasted across the 5 days of evaluation, while neutrophils gradually infiltrated the immune clusters at later time points. Using a combination of neutrophil- and macrophage-deficient (or depleted) fish, the authors found evidence that the killing of CEA10 (but not Af293) isolates was largely attributed to neutrophils, rather than macrophages. Moreover, macrophage-deficient fish show 60% more efficient killing of CEA10 than immunocompetent fish at 2 dpi, which suggests that macrophages may provide a protective niche for *A. fumigatus* to evade neutrophil-mediated killing. Importantly, in macrophage-depleted zebrafish, a larger amount of germination is observed for both strains, suggesting that macrophages have germination-inhibitory activity that may mask *A. fumigatus* from the hyphal-specific antifungal potency of neutrophils [18]. Together, in vivo imaging in zebrafish was able to show that a germination rate can alter the recruitment and antifungal activity of immune cells while also being modulated by macrophages. 

The authors were able to further link germination to neutrophil killing activity, in vivo, using uridine-uracil auxotrophs (pyrG mutants) with known germination defects. In macrophage-deficient hosts, neutrophils were able to clear ~85% of CEA10 by 1 dpi; however, for the germination-defective strain, the clearance was lower with only ~40% by 1 dpi, a difference that was maintained throughout later time points of infection. Together, these results suggest that germination of *A. fumigatus* is key to neutrophil-mediated killing and explains why faster germinating strains are more effectively cleared. Based on all of these findings, it seems like the role of germination in virulence is likely a balancing act between host invasion and host evasion, where it is beneficial to germinate fast enough to become a resistant, vegetatively growing cells (hyphae), while also trying to avoid immune response, primarily neutrophil-mediated killing. This does put forth an interesting question: in which scenarios would it be beneficial to inhibit germination to minimise host damage or, rather, to increase germination to activate host defences? Variations in disease presentation (IA, ABPA and CPA) could be due to variations of germination kinetics in the presence of different host immune environments, and more research into understanding the kinetics of germination is needed before we can successfully use it as a target in the prevention of aspergillosis.

Zebrafish offer many advantages for in vivo imaging [171,172,174,175]. Due to their transparent nature, zebrafish larvae provide an infection model that allows for non-invasive cellular imaging in the same live hosts over multiple days of infection (longitudinal studies). With the development of genetically engineered fluorescent fungal strains and transgenic fish with fluorescently labelled immune cell populations, the complex host–pathogen interactions occurring during the early stages of infection can be investigated. Notably, temporal assessment of the same fungal clusters and cells over time can be performed. The high resolution of such imaging not only allows for characterisation of immune recruitment, but also for changes in fungal morphology. For instance, using this zebrafish model, a comparison of *A. fumigatus* and *A. niger* indicates that *A. fumigatus* is more readily taken up by macrophages than *A. niger* and that *A. fumigatus* readily germinates inside macrophages, while *A. niger* depends on extracellular germination to establish an infection [173]. These differences in intracellular versus extracellular germination are likely key to understanding the disease kinetics of these opportunistic pathogens. The use of this model can be expanded to *A. fumigatus* mutants with cell wall defects and transgenic zebrafish lacking specific immune cell receptors to explore the role of specific fungal epitopes and host receptors in morphotype-specific immune cell recruitment and activity. An additional benefit of this model is that, because live imaging can be performed for the whole organism, collection and imaging bias are avoided. For infection with *A. fumigatus*, this infection model also only requires a very small infectious inoculum (only 10–100 conidia), while murine models require significantly more (on occasions up to 10^7^ conidia) [171]. This is not only more physiologically relevant to human exposure dosages from the environment [1], but it is also more practical, for example, when using strains that have poor conidiation, thereby requiring larger culturing volumes to harvest a sufficient number of spores. By replacing mice with larval zebrafish, partial replacement is also achieved, hence representing an improvement in line with the principle of the 3Rs (replacement, reduction and refinement), aimed at performing more humane animal research. However, there are some notable drawbacks of using this infection model. The biggest limitation is that infections are performed by injection into the hindbrain, thus not mimicking human infections, which generally start via spore inhalation. Alterations in infection route might affect the nutritional and immunological context in which infection takes place, bypassing the complex interaction with different host cell types that ultimately contributes to disease outcome. It is also important to note that zebrafish are kept at 28 °C, which is a significantly lower temperature than that of mammalian hosts (37 °C), with possible implications for the growth, behaviour and virulence of *A. fumigatus* [176]. However, the development of an Arabian killifish (*Aphanius dispar*) model holds promise for infection models, as this fish can function physiologically at 37 °C [177]. Finally, zebrafish larvae only have an innate immune system, so one cannot assess the role of the adaptive immune system in this model; however, the lack of an adaptive immune system ultimately provides a simplified host environment, which can be exploited to understand in-depth the innate immune system’s role in infection. The limitations listed here do not overshadow the incredible benefits of this model, which is proving valuable in the study of in vivo host–pathogen interactions. 

## 6. Conclusions

State-of-the-art technologies for imaging of the host–pathogen interaction during infection have helped to derive novel insights into *A. fumigatus* pathogenesis and host response, as highlighted by the four separate examples provided here, which illustrate how these techniques have been used to answer key questions in the field. We placed these questions in the context of known research and highlighted key studies that have used these techniques to make significant leaps in our understanding. While each of the techniques used has its advantages and disadvantages (see Table 1), different questions require different approaches. Each of these techniques is, and will be, essential to developing a more complete understanding of the mechanisms underlying *A. fumigatus* pathogenesis and host response to inform the use of current and novel antifungal therapeutics and diagnostics and minimise the high mortality caused by aspergillosis.

## Figures and Tables

**Table 1:** Summary of advantages and disadvantages associated with different in vivo imaging techniques discussed in this review.

	Light Sheet Fluorescence Microscopy (LSFM)	Fluorescence Tomography/Bioluminescence Tomography	Imaging Flow Cytometry (IFC)	Zebrafish Model of Aspergillosis
**Advantages**	− Maintains 3D lung architecture and provides spatial-anatomical context of an infected lung. − High enough resolution to image entire lung lobes at cellular resolution allows for the characterisation of fungal growth and host immune recruitment.	− Allows for longitudinal studies of the same mice over time. − Provides improvement in the 3Rs (reduction). − Maintains 3D lung architecture and provides spatial-anatomical context of an infected lung.	− High resolution enables the assessment of host–pathogen interactions with single-cell resolution, even when these interactions are relatively rare events. − Large number of cells analysed.	− High resolution live cell 4D imaging to monitor individual fungal cell morphology and immune cell recruitment to specific infection sites. − Allows for longitudinal studies of the same zebrafish over time. − Requires a relatively small inoculum if limited samples are an issue. − Provides an improvement in the 3Rs (partial replacement). − Lack of adaptive immune system provides a simpler system to characterise the innate immune system.
**Disadvantages**	− Laborious and expensive sample preparation that requires fixation. − Not feasible to do longitudinal studies of the same host over time.	− Quantifying *A. fumigatus* is hampered by the use of luminescence strains affected by local oxygen levels and poor resolution. − Lower resolution than other in vivo imaging techniques.	− Requires cells to be in suspension, thus does not provide spatial-anatomical context of an infected lung. − Not feasible to do longitudinal studies of the same host over time.	− Infectious route does not mimic natural infection and ignores lung environment. − Host temperature much lower than mammalian host. − Inability to assess role of adaptive immune system.
**Processing**	− Requires harvesting of lung lobules for whole lung clearing.	− Imaging of live mice (no processing). − Requires anesthetising mice prior to placing into imaging chamber.	− Analysed cells have to be in suspension (infected organs need to be extracted and dissociated).	− In live zebrafish (no processing). − Requires anaesthetising fish and low-melting agarose preparation.
**Key Study**	Amich et al. (2020) [15]	Gresnigt et al. (2016) [16]	Bertuzzi et al. (2022) [17]	Rosowski et al. (2018) [18]
**Alternatives**	Histology: labour intensive and practically unfeasible for whole lungs, potentially resulting in sampling bias;Confocal laser-scanning microscopy: requires same clearing process as LSFM and has similar disadvantages but may provide higher spatial resolution; however, the imaging depth is limited to approx. 1mm fluorescence signal-pending;ImmunoPET/MRI: similar to fluorescence tomography but primarily being explored as a diagnostic tool.			

## Data Availability

Not applicable.

## References

[1] O’Gorman C. (2021). Airborne *Aspergillus fumigatus* conidia: A risk factor for aspergillosis. Fungal Biol. Rev..

[2] Brown G.D., Denning D.W., Gow N.A., Levitz S.M., Netea M.G., White T.C. (2012). Hidden killers: Human fungal infections. Sci. Transl. Med..

[3] Bongomin F., Gago S., Oladele R.O., Denning D.W. (2017). Global and multi-national prevalence of fungal diseases-estimate precision. J. Fungi.

[4] Guinea J., Torres-Narbona M., Gijón P., Muñoz P., Pozo F., Peláez T., de Miguel J., Bouza E. (2010). Pulmonary aspergillosis in patients with chronic obstructive pulmonary disease: Incidence, risk factors, and outcome. Clin. Microbiol. Infect..

[5] Hammond E.E., McDonald C.S., Vestbo J., Denning D.W. (2020). The global impact of *Aspergillus* infection on COPD. BMC Pulm. Med..

[6] Knutsen A.P., Slavin R.G. (2011). Allergic bronchopulmonary aspergillosis in asthma and cystic fibrosis. Clin. Dev. Immunol..

[7] Denning D.W., Bromley M.J. (2015). Infectious Disease. How to bolster the antifungal pipeline. Science.

[8] Toda M., Beer K.D., Kuivila K.M., Chiller T.M., Jackson B.R. (2021). Trends in agricultural triazole fungicide use in the United States, 1992-2016 and possible implications for antifungal-resistant fungi in human disease. Environ. Health Perspect..

[9] Verweij P.E., Lucas J.A., Arendrup M.C., Bowyer P., Brinkmann A.J., Denning D.W., Dyer P.S., Fisher M.C., Geenen P.L., Gisi U. (2020). The one health problem of azole resistance in *Aspergillus fumigatus*: Current insights and future research agenda. Fungal Biol. Rev..

[10] Gunzer M., Thornton C.R., Beziere N. (2020). Advances in the in vivo molecular imaging of invasive aspergillosis. J. Fungi.

[11] Latgé J.P., Chamilos G. (2019). *Aspergillus fumigatus* and Aspergillosis in 2019. Clin. Microbiol. Rev..

[12] Mackel J.J., Steele C. (2019). Host defense mechanisms against *Aspergillus fumigatus* lung colonization and invasion. Curr. Opin. Microbiol..

[13] van de Veerdonk F.L., Gresnigt M.S., Romani L., Netea M.G., Latgé J.P. (2017). *Aspergillus fumigatus* morphology and dynamic host interactions. Nat. Rev. Microbiol..

[14] Latgé J.P., Beauvais A., Chamilos G. (2017). The cell wall of the human fungal pathogen *Aspergillus fumigatus*: Biosynthesis, organization, immune response, and virulence. Annu. Rev. Microbiol..

[15] Amich J., Mokhtari Z., Strobel M., Vialetto E., Sheta D., Yu Y., Hartweg J., Kalleda N., Jarick K.J., Brede C. (2020). Three-dimensional light sheet fluorescence microscopy of lungs to dissect local host immune-*Aspergillus fumigatus* interactions. mBio.

[16] Gresnigt M.S., Rekiki A., Rasid O., Savers A., Jouvion G., Dannaoui E., Parlato M., Fitting C., Brock M., Cavaillon J.M. (2016). Reducing hypoxia and inflammation during invasive pulmonary aspergillosis by targeting the Interleukin-1 receptor. Sci. Rep..

[17] Bertuzzi M., Howell G.J., Thomson D.D., Fortune-Grant R., Moeslinger A., Dancer P., Van Rhijn N., Motsi N., Du X., Codling A. Epithelial uptake of *Aspergillus fumigatus* drives efficient fungal clearance in vivo and is aberrant in Chronic Obstructive Pulmonary Disease (COPD). bioRxiv.

[18] Rosowski E.E., Raffa N., Knox B.P., Golenberg N., Keller N.P., Huttenlocher A. (2018). Macrophages inhibit *Aspergillus fumigatus* germination and neutrophil-mediated fungal killing. PLoS Pathog..

[19] American Society for Microbiology (2019). One Health: Fungal Pathogens of Humans, Animals, and Plants: Report on an American Academy of Microbiology Colloquium Held in Washington, DC, USA on 18 October 2017.

[20] Nicod L.P. (2005). Lung defences: An overview. Eur. Respir. Rev..

[21] Bertuzzi M., Hayes G.E., Icheoku U.J., van Rhijn N., Denning D.W., Osherov N., Bignell E.M. (2018). Anti-*Aspergillus* activities of the respiratory epithelium in health and disease. J. Fungi.

[22] McCormick A., Loeffler J., Ebel F. (2010). *Aspergillus fumigatus*: Contours of an opportunistic human pathogen. Cell. Microbiol..

[23] Latgé J.P. (1999). *Aspergillus fumigatus* and aspergillosis. Clin. Microbiol. Rev..

[24] Brakhage A.A., Langfelder K. (2002). Menacing mold: The molecular biology of *Aspergillus fumigatus*. Annu. Rev. Microbiol..

[25] Dagenais T.R., Keller N.P. (2009). Pathogenesis of *Aspergillus fumigatus* in invasive aspergillosis. Clin. Microbiol. Rev..

[26] Brede C., Friedrich M., Jordán-Garrote A.L., Riedel S.S., Bäuerlein C.A., Heinze K.G., Bopp T., Schulz S., Mottok A., Kiesel C. (2012). Mapping immune processes in intact tissues at cellular resolution. J. Clin. Investig..

[27] Rolle A.M., Hasenberg M., Thornton C.R., Solouk-Saran D., Männ L., Weski J., Maurer A., Fischer E., Spycher P.R., Schibli R. (2016). ImmunoPET/MR imaging allows specific detection of *Aspergillus fumigatus* lung infection in vivo. Proc. Natl. Acad. Sci. USA.

[28] Soutiere S.E., Tankersley C.G., Mitzner W. (2004). Differences in alveolar size in inbred mouse strains. Respir. Physiol. Neurobiol..

[29] Knust J., Ochs M., Gundersen H.J., Nyengaard J.R. (2009). Stereological estimates of alveolar number and size and capillary length and surface area in mice lungs. Anat. Rec..

[30] Mercer R.R., Russell M.L., Crapo J.D. (1994). Alveolar septal structure in different species. J. Appl. Physiol..

[31] Bertuzzi M., Schrettl M., Alcazar-Fuoli L., Cairns T.C., Muñoz A., Walker L.A., Herbst S., Safari M., Cheverton A.M., Chen D. (2014). The pH-responsive PacC transcription factor of *Aspergillus fumigatus* governs epithelial entry and tissue invasion during pulmonary aspergillosis. PLoS Pathog..

[32] Croft C.A., Culibrk L., Moore M.M., Tebbutt S.J. (2016). Interactions of *Aspergillus fumigatus* conidia with airway epithelial cells: A Critical Review. Front. Microbiol..

[33] Geiser M., Leupin N., Maye I., Hof V.I., Gehr P. (2000). Interaction of fungal spores with the lungs: Distribution and retention of inhaled puffball (*Calvatia excipuliformis*) spores. J. Allergy Clin. Immunol..

[34] Rammaert B., Jouvion G., de Chaumont F., Garcia-Hermoso D., Szczepaniak C., Renaudat C., Olivo-Marin J.C., Chrétien F., Dromer F., Bretagne S. (2015). Absence of fungal spore internalization by bronchial epithelium in mouse models evidenced by a new bioimaging approach and transmission electronic microscopy. Am. J. Pathol..

[35] Secondo L.E., Sagona J.A., Calderón L., Wang Z., Plotnik D., Senick J., Sorensen-Allacci M., Wener R., Andrews C.J., Mainelis G. (2021). Estimating lung deposition of fungal spores using actual airborne spore concentrations and physiological data. Environ. Sci. Technol..

[36] Sagona J., Secondo L., Mainelis G. (2020). Comparison of two models to estimate deposition of fungi and bacteria in the human respiratory tract. Atmosphere.

[37] Cho S., Seo S.-C., Schmechel D., Grinshpun S., Reponen T. (2005). Aerodynamic characteristic and respiratory deposition of fungal particles. Atmos. Environ..

[38] Madhwal S., Prabhu V., Sundriyal S., Shridhar V. (2019). Distribution, characterization and health risk assessment of size fractionated bioaerosols at an open landfill site in Dehradun, India. Atmos. Pollut. Res..

[39] Reponen T. (1995). Aerodynamic diameters and respiratory deposition estimates of viable fungal particles in mold problem dwellings. Aerosol Sci. Technol..

[40] Bogorodskiy A.O., Bolkhovitina E.L., Gensch T., Troyanova N.I., Mishin A.V., Okhrimenko I.S., Braun A., Spies E., Gordeliy V.I., Sapozhnikov A.M. (2020). Murine intraepithelial dendritic cells interact with phagocytic cells during *Aspergillus fumigatus*-induced inflammation. Front. Immunol..

[41] Maslov I.V., Bogorodskiy A.O., Pavelchenko M.V., Zykov I.O., Troyanova N.I., Borshchevskiy V.I., Shevchenko M.A. (2021). Confocal laser scanning microscopy-based quantitative analysis of *Aspergillus fumigatus* conidia distribution in whole-mount optically cleared mouse lung. J. Vis. Exp. JoVE.

[42] King J., Brunel S.F., Warris A. (2016). *Aspergillus* infections in cystic fibrosis. J. Infect..

[43] Belchi F., Pirashvili M., Conway J., Bennett M., Djukanovic R., Brodzki J. (2018). Lung topology characteristics in patients with chronic obstructive pulmonary disease. Sci. Rep..

[44] Meyerholz D.K., Stoltz D.A., Namati E., Ramachandran S., Pezzulo A.A., Smith A.R., Rector M.V., Suter M.J., Kao S., McLennan G. (2010). Loss of cystic fibrosis transmembrane conductance regulator function produces abnormalities in tracheal development in neonatal pigs and young children. Am. J. Respir. Crit. Care Med..

[45] Semaniakou A., Croll R.P., Chappe V. (2019). Animal models in the pathophysiology of cystic fibrosis. Front. Pharmacol..

[46] Sugui J.A., Kwon-Chung K.J., Juvvadi P.R., Latgé J.P., Steinbach W.J. (2014). *Aspergillus fumigatus* and related species. Cold Spring Harb. Perspect. Med..

[47] Grahl N., Puttikamonkul S., Macdonald J.M., Gamcsik M.P., Ngo L.Y., Hohl T.M., Cramer R.A. (2011). In vivo hypoxia and a fungal alcohol dehydrogenase influence the pathogenesis of invasive pulmonary aspergillosis. PLoS Pathog..

[48] Warn P.A., Sharp A., Guinea J., Denning D.W. (2004). Effect of hypoxic conditions on in vitro susceptibility testing of amphotericin B, itraconazole and micafungin against *Aspergillus* and *Candida*. J. Antimicrob. Chemother..

[49] West J.B. (1985). Respiratory Physiology—The Essentials.

[50] Simmen H.P., Battaglia H., Giovanoli P., Blaser J. (1994). Analysis of pH, pO2 and pCO2 in drainage fluid allows for rapid detection of infectious complications during the follow-up period after abdominal surgery. Infection.

[51] Simmen H.P., Blaser J. (1993). Analysis of pH and pO2 in abscesses, peritoneal fluid, and drainage fluid in the presence or absence of bacterial infection during and after abdominal surgery. Am. J. Surg..

[52] Grahl N., Kowalski C.H., Cramer R.A. (2021). Detection of low oxygen microenvironments in a murine model of invasive pulmonary aspergillosis using Pimonidazole. Methods Mol. Biol..

[53] Grahl N., Cramer R.A. (2010). Regulation of hypoxia adaptation: An overlooked virulence attribute of pathogenic fungi?. Med. Mycol..

[54] Tarrand J.J., Han X.Y., Kontoyiannis D.P., May G.S. (2005). *Aspergillus* hyphae in infected tissue: Evidence of physiologic adaptation and effect on culture recovery. J. Clin. Microbiol..

[55] Brock M., Jouvion G., Droin-Bergère S., Dussurget O., Nicola M.A., Ibrahim-Granet O. (2008). Bioluminescent *Aspergillus fumigatus*, a new tool for drug efficiency testing and in vivo monitoring of invasive aspergillosis. Appl. Environ. Microbiol..

[56] Willger S.D., Puttikamonkul S., Kim K.H., Burritt J.B., Grahl N., Metzler L.J., Barbuch R., Bard M., Lawrence C.B., Cramer R.A. (2008). A sterol-regulatory element binding protein is required for cell polarity, hypoxia adaptation, azole drug resistance, and virulence in *Aspergillus fumigatus*. PLoS Pathog..

[57] Ibrahim-Granet O., Jouvion G., Hohl T.M., Droin-Bergère S., Philippart F., Kim O.Y., Adib-Conquy M., Schwendener R., Cavaillon J.M., Brock M. (2010). In vivo bioluminescence imaging and histopathopathologic analysis reveal distinct roles for resident and recruited immune effector cells in defense against invasive aspergillosis. BMC Microbiol..

[58] Hsu J.L., Khan M.A., Sobel R.A., Jiang X., Clemons K.V., Nguyen T.T., Stevens D.A., Martinez M., Nicolls M.R. (2013). *Aspergillus fumigatus* invasion increases with progressive airway ischemia. PLoS ONE.

[59] Kowalski C.H., Beattie S.R., Fuller K.K., McGurk E.A., Tang Y.W., Hohl T.M., Obar J.J., Cramer R.A. (2016). Heterogeneity among isolates reveals that fitness in low oxygen correlates with *Aspergillus fumigatus* virulence. mBio.

[60] Dhingra S., Buckey J.C., Cramer R.A. (2018). Hyperbaric oxygen reduces *Aspergillus fumigatus* proliferation in vitro and influences in vivo disease outcomes. Antimicrob. Agents Chemother..

[61] Willger S.D., Cornish E.J., Chung D., Fleming B.A., Lehmann M.M., Puttikamonkul S., Cramer R.A. (2012). Dsc orthologs are required for hypoxia adaptation, triazole drug responses, and fungal virulence in *Aspergillus fumigatus*. Eukaryot. Cell.

[62] Chung D., Barker B.M., Carey C.C., Merriman B., Werner E.R., Lechner B.E., Dhingra S., Cheng C., Xu W., Blosser S.J. (2014). ChIP-seq and in vivo transcriptome analyses of the *Aspergillus fumigatus* SREBP SrbA reveals a new regulator of the fungal hypoxia response and virulence. PLoS Pathog..

[63] Blosser S.J., Merriman B., Grahl N., Chung D., Cramer R.A. (2014). Two C4-sterol methyl oxidases (Erg25) catalyse ergosterol intermediate demethylation and impact environmental stress adaptation in *Aspergillus fumigatus*. Microbiology.

[64] Vaknin Y., Hillmann F., Iannitti R., Ben Baruch N., Sandovsky-Losica H., Shadkchan Y., Romani L., Brakhage A., Kniemeyer O., Osherov N. (2016). Identification and characterization of a novel *Aspergillus fumigatus* rhomboid family putative protease, RbdA, involved in hypoxia sensing and virulence. Infect. Immun..

[65] Kroll K., Shekhova E., Mattern D.J., Thywissen A., Jacobsen I.D., Strassburger M., Heinekamp T., Shelest E., Brakhage A.A., Kniemeyer O. (2016). The hypoxia-induced dehydrogenase HorA is required for coenzyme Q10 biosynthesis, azole sensitivity and virulence of *Aspergillus fumigatus*. Mol. Microbiol..

[66] Barker B.M., Kroll K., Vödisch M., Mazurie A., Kniemeyer O., Cramer R.A. (2012). Transcriptomic and proteomic analyses of the *Aspergillus fumigatus* hypoxia response using an oxygen-controlled fermenter. BMC Genom..

[67] Losada L., Barker B.M., Pakala S., Pakala S., Joardar V., Zafar N., Mounaud S., Fedorova N., Nierman W.C., Cramer R.A. (2014). Large-scale transcriptional response to hypoxia in *Aspergillus fumigatus* observed using RNAseq identifies a novel hypoxia regulated ncRNA. Mycopathologia.

[68] Kale S.D., Ayubi T., Chung D., Tubau-Juni N., Leber A., Dang H.X., Karyala S., Hontecillas R., Lawrence C.B., Cramer R.A. (2017). Modulation of immune signaling and metabolism highlights host and fungal transcriptional responses in mouse models of invasive pulmonary aspergillosis. Sci. Rep..

[69] Vödisch M., Scherlach K., Winkler R., Hertweck C., Braun H.P., Roth M., Haas H., Werner E.R., Brakhage A.A., Kniemeyer O. (2011). Analysis of the *Aspergillus fumigatus* proteome reveals metabolic changes and the activation of the pseurotin A biosynthesis gene cluster in response to hypoxia. J. Proteome Res..

[70] Kroll K., Pähtz V., Hillmann F., Vaknin Y., Schmidt-Heck W., Roth M., Jacobsen I.D., Osherov N., Brakhage A.A., Kniemeyer O. (2014). Identification of hypoxia-inducible target genes of *Aspergillus fumigatus* by transcriptome analysis reveals cellular respiration as an important contributor to hypoxic survival. Eukaryot. Cell.

[71] Shekhova E., Ivanova L., Krüger T., Stroe M.C., Macheleidt J., Kniemeyer O., Brakhage A.A. (2019). Redox proteomic analysis reveals oxidative modifications of proteins by increased levels of intracellular reactive oxygen species during hypoxia adaptation of *Aspergillus fumigatus*. Proteomics.

[72] Rees C.A., Stefanuto P.H., Beattie S.R., Bultman K.M., Cramer R.A., Hill J.E. (2017). Sniffing out the hypoxia volatile metabolic signature of *Aspergillus fumigatus*. J. Breath Res..

[73] Binder U., Maurer E., Lackner M., Lass-Flörl C. (2015). Effect of reduced oxygen on the antifungal susceptibility of clinically relevant aspergilli. Antimicrob. Agents Chemother..

[74] Kowalski C.H., Morelli K.A., Schultz D., Nadell C.D., Cramer R.A. (2020). Fungal biofilm architecture produces hypoxic microenvironments that drive antifungal resistance. Proc. Natl. Acad. Sci. USA.

[75] Maurer E., Aigner M., Lass-Flörl C., Binder U. (2019). Hypoxia decreases diagnostic biomarkers for Aspergillosis in vitro. J. Fungi.

[76] Wezensky S.J., Cramer R.A. (2011). Implications of hypoxic microenvironments during invasive aspergillosis. Med. Mycol..

[77] Grahl N., Shepardson K.M., Chung D., Cramer R.A. (2012). Hypoxia and fungal pathogenesis: To air or not to air?. Eukaryot. Cell.

[78] Hillmann F., Shekhova E., Kniemeyer O. (2015). Insights into the cellular responses to hypoxia in filamentous fungi. Curr. Genet..

[79] Chung H., Lee Y.H. (2020). Hypoxia: A double-edged sword during fungal pathogenesis?. Front. Microbiol..

[80] Bao B., Groves K., Zhang J., Handy E., Kennedy P., Cuneo G., Supuran C.T., Yared W., Rajopadhye M., Peterson J.D. (2012). In vivo imaging and quantification of carbonic anhydrase IX expression as an endogenous biomarker of tumor hypoxia. PLoS ONE.

[81] Carvalho A., Cunha C., Iannitti R.G., De Luca A., Giovannini G., Bistoni F., Romani L. (2012). Inflammation in aspergillosis: The good, the bad, and the therapeutic. Ann. N. Y. Acad. Sci..

[82] Fröhlich S., Boylan J., McLoughlin P. (2013). Hypoxia-induced inflammation in the lung: A potential therapeutic target in acute lung injury?. Am. J. Respir. Cell Mol. Biol..

[83] Rider P., Kaplanov I., Romzova M., Bernardis L., Braiman A., Voronov E., Apte R.N. (2012). The transcription of the alarmin cytokine interleukin-1 alpha is controlled by hypoxia inducible factors 1 and 2 alpha in hypoxic cells. Front. Immunol..

[84] Caffrey A.K., Lehmann M.M., Zickovich J.M., Espinosa V., Shepardson K.M., Watschke C.P., Hilmer K.M., Thammahong A., Barker B.M., Rivera A. (2015). IL-1α signaling is critical for leukocyte recruitment after pulmonary *Aspergillus fumigatus* challenge. PLoS Pathog..

[85] Gabay C., Lamacchia C., Palmer G. (2010). IL-1 pathways in inflammation and human diseases. Nat. Rev. Rheumatol..

[86] Shepardson K.M., Ngo L.Y., Aimanianda V., Latgé J.P., Barker B.M., Blosser S.J., Iwakura Y., Hohl T.M., Cramer R.A. (2013). Hypoxia enhances innate immune activation to *Aspergillus fumigatus* through cell wall modulation. Microbes Infect..

[87] Fliesser M., Wallstein M., Kurzai O., Einsele H., Löffler J. (2016). Hypoxia attenuates anti-*Aspergillus fumigatus* immune responses initiated by human dendritic cells. Mycoses.

[88] Hutchens M., Luker G.D. (2007). Applications of bioluminescence imaging to the study of infectious diseases. Cell. Microbiol..

[89] Contag C.H., Contag P.R., Mullins J.I., Spilman S.D., Stevenson D.K., Benaron D.A. (1995). Photonic detection of bacterial pathogens in living hosts. Mol. Microbiol..

[90] Campbell A.K. (1988). Chemiluminescence. Principles and Applications in Biology and Medicine.

[91] Robertson J.B., Stowers C.C., Boczko E., Johnson C.H. (2008). Real-time luminescence monitoring of cell-cycle and respiratory oscillations in yeast. Proc. Natl. Acad. Sci. USA.

[92] Hastings J.W., McElroy W.D., Coulombre J. (1953). The effect of oxygen upon the immobilization reaction in firefly luminescence. J. Cell. Comp. Physiol..

[93] Poelmans J., Himmelreich U., Vanherp L., Zhai L., Hillen A., Holvoet B., Belderbos S., Brock M., Maertens J., Vande Velde G. (2018). A multimodal imaging approach enables in vivo assessment of antifungal treatment in a mouse model of invasive pulmonary aspergillosis. Antimicrob. Agents Chemother..

[94] Davies G., Rolle A.M., Maurer A., Spycher P.R., Schillinger C., Solouk-Saran D., Hasenberg M., Weski J., Fonslet J., Dubois A. (2017). Towards translational immunoPET/MR imaging of invasive pulmonary aspergillosis: The humanised monoclonal antibody JF5 detects *Aspergillus* lung infections in vivo. Theranostics.

[95] Thornton C.R. (2018). Molecular imaging of invasive pulmonary aspergillosis using immunoPET/MRI: The future looks bright. Front. Microbiol..

[96] Henneberg S., Hasenberg A., Maurer A., Neumann F., Bornemann L., Gonzalez-Menendez I., Kraus A., Hasenberg M., Thornton C.R., Pichler B.J. (2021). Antibody-guided in vivo imaging of *Aspergillus fumigatus* lung infections during antifungal azole treatment. Nat. Commun..

[97] Kent B.D., Mitchell P.D., McNicholas W.T. (2011). Hypoxemia in patients with COPD: Cause, effects, and disease progression. Int. J. Chronic Obstr. Pulm. Dis..

[98] Montgomery S.T., Mall M.A., Kicic A., Stick S.M., Arest C.F. (2017). Hypoxia and sterile inflammation in cystic fibrosis airways: Mechanisms and potential therapies. Eur. Respir. J..

[99] Fritzsching B., Zhou-Suckow Z., Trojanek J.B., Schubert S.C., Schatterny J., Hirtz S., Agrawal R., Muley T., Kahn N., Sticht C. (2015). Hypoxic epithelial necrosis triggers neutrophilic inflammation via IL-1 receptor signaling in cystic fibrosis lung disease. Am. J. Respir. Crit. Care Med..

[100] Bignell E., Negrete-Urtasun S., Calcagno A.M., Haynes K., Arst H.N., Rogers T. (2005). The *Aspergillus* pH-responsive transcription factor PacC regulates virulence. Mol. Microbiol..

[101] Barda O., Maor U., Sadhasivam S., Bi Y., Zakin V., Prusky D., Sionov E. (2020). The pH-responsive transcription factor PacC governs pathogenicity and ochratoxin A biosynthesis in *Aspergillus carbonarius*. Front. Microbiol..

[102] Amich J., Vicentefranqueira R., Mellado E., Ruiz-Carmuega A., Leal F., Calera J.A. (2014). The ZrfC alkaline zinc transporter is required for *Aspergillus fumigatus* virulence and its growth in the presence of the Zn/Mn-chelating protein calprotectin. Cell. Microbiol..

[103] Amich J., Calera J.A. (2014). Zinc acquisition: A key aspect in *Aspergillus fumigatus* virulence. Mycopathologia.

[104] Berezin M.Y., Guo K., Akers W., Northdurft R.E., Culver J.P., Teng B., Vasalatiy O., Barbacow K., Gandjbakhche A., Griffiths G.L. (2011). Near-infrared fluorescence lifetime pH-sensitive probes. Biophys. J..

[105] Ghosh S.K., Kim P., Zhang X.A., Yun S.H., Moore A., Lippard S.J., Medarova Z. (2010). A novel imaging approach for early detection of prostate cancer based on endogenous zinc sensing. Cancer Res..

[106] Schaffner A., Douglas H., Braude A. (1982). Selective protection against conidia by mononuclear and against mycelia by polymorphonuclear phagocytes in resistance to *Aspergillus*. Observations on these two lines of defense in vivo and in vitro with human and mouse phagocytes. J. Clin. Investig..

[107] Ibrahim-Granet O., Philippe B., Boleti H., Boisvieux-Ulrich E., Grenet D., Stern M., Latgé J.P. (2003). Phagocytosis and intracellular fate of *Aspergillus fumigatus* conidia in alveolar macrophages. Infect. Immun..

[108] Philippe B., Ibrahim-Granet O., Prévost M.C., Gougerot-Pocidalo M.A., Sanchez Perez M., Van der Meeren A., Latgé J.P. (2003). Killing of *Aspergillus fumigatus* by alveolar macrophages is mediated by reactive oxidant intermediates. Infect. Immun..

[109] Volling K., Thywissen A., Brakhage A.A., Saluz H.P. (2011). Phagocytosis of melanized *Aspergillus* conidia by macrophages exerts cytoprotective effects by sustained PI3K/Akt signalling. Cell. Microbiol..

[110] Mircescu M.M., Lipuma L., van Rooijen N., Pamer E.G., Hohl T.M. (2009). Essential role for neutrophils but not alveolar macrophages at early time points following *Aspergillus fumigatus* infection. J. Infect. Dis..

[111] Tanaka R.J., Boon N.J., Vrcelj K., Nguyen A., Vinci C., Armstrong-James D., Bignell E. (2015). In silico modeling of spore inhalation reveals fungal persistence following low dose exposure. Sci. Rep..

[112] Pollmächer J., Figge M.T. (2014). Agent-based model of human alveoli predicts chemotactic signaling by epithelial cells during early *Aspergillus fumigatus* infection. PLoS ONE.

[113] Bonnett C.R., Cornish E.J., Harmsen A.G., Burritt J.B. (2006). Early neutrophil recruitment and aggregation in the murine lung inhibit germination of *Aspergillus fumigatus* conidia. Infect. Immun..

[114] Feldman M.B., Dutko R.A., Wood M.A., Ward R.A., Leung H.M., Snow R.F., De La Flor D.J., Yonker L.M., Reedy J.L., Tearney G.J. (2020). *Aspergillus fumigatus* cell wall promotes apical airway epithelial recruitment of human neutrophils. Infect. Immun..

[115] Crapo J.D., Barry B.E., Gehr P., Bachofen M., Weibel E.R. (1982). Cell number and cell characteristics of the normal human lung. Am. Rev. Respir. Dis..

[116] Bertuzzi M., Hayes G.E., Bignell E.M. (2019). Microbial uptake by the respiratory epithelium: Outcomes for host and pathogen. FEMS Microbiol. Rev..

[117] Ewald J., Rivieccio F., Radosa L., Schuster S., Brakhage A.A., Kaleta C. (2021). Dynamic optimization reveals alveolar epithelial cells as key mediators of host defense in invasive aspergillosis. PLoS Comput. Biol..

[118] Blickensdorf M., Timme S., Figge M.T. (2020). Hybrid agent-based modeling of *Aspergillus fumigatus* Infection to quantitatively investigate the role of Pores of Kohn in human alveoli. Front. Microbiol..

[119] Pollmächer J., Figge M.T. (2015). Deciphering chemokine properties by a hybrid agent-based model of *Aspergillus fumigatus* infection in human alveoli. Front. Microbiol..

[120] Wasylnka J.A., Moore M.M. (2002). Uptake of *Aspergillus fumigatus* conidia by phagocytic and nonphagocytic cells in vitro: Quantitation using strains expressing green fluorescent protein. Infect. Immun..

[121] Han X., Yu R., Zhen D., Tao S., Schmidt M., Han L. (2011). β-1,3-Glucan-induced host phospholipase D activation is involved in *Aspergillus fumigatus* internalization into type II human pneumocyte A549 cells. PLoS ONE.

[122] Wasylnka J.A., Moore M.M. (2003). *Aspergillus fumigatus* conidia survive and germinate in acidic organelles of A549 epithelial cells. J. Cell Sci..

[123] Oosthuizen J.L., Gomez P., Ruan J., Hackett T.L., Moore M.M., Knight D.A., Tebbutt S.J. (2011). Dual organism transcriptomics of airway epithelial cells interacting with conidia of *Aspergillus fumigatus*. PLoS ONE.

[124] Gomez P., Hackett T.L., Moore M.M., Knight D.A., Tebbutt S.J. (2010). Functional genomics of human bronchial epithelial cells directly interacting with conidia of *Aspergillus fumigatus*. BMC Genom..

[125] Paris S., Boisvieux-Ulrich E., Crestani B., Houcine O., Taramelli D., Lombardi L., Latgé J.P. (1997). Internalization of *Aspergillus fumigatus* conidia by epithelial and endothelial cells. Infect. Immun..

[126] Seidel C., Moreno-Velásquez S.D., Ben-Ghazzi N., Gago S., Read N.D., Bowyer P. (2020). Phagolysosomal survival enables non-lytic hyphal escape and ramification through lung epithelium during *Aspergillus fumigatus* infection. Front. Microbiol..

[127] Amitani R., Kawanami R. (2009). Interaction of *Aspergillus* with human respiratory mucosa: A study with organ culture model. Med. Mycol..

[128] Ben-Ghazzi N., Moreno-Velásquez S., Seidel C., Thomson D., Denning D.W., Read N.D., Bowyer P., Gago S. (2021). Characterisation of *Aspergillus fumigatus* endocytic trafficking within airway epithelial cells using high-resolution automated quantitative confocal microscopy. J. Fungi.

[129] Keizer E.M., Wösten H., de Cock H. (2020). EphA2-dependent internalization of *A. fumigatus* conidia in A549 lung cells is modulated by DHN-melanin. Front. Microbiol..

[130] Fernandes J., Hamidi F., Leborgne R., Beau R., Castier Y., Mordant P., Boukkerou A., Latgé J.P., Pretolani M. (2018). Penetration of the human pulmonary epithelium by *Aspergillus fumigatus* hyphae. J. Infect. Dis..

[131] Bidula S., Kenawy H., Ali Y.M., Sexton D., Schwaeble W.J., Schelenz S. (2013). Role of ficolin-A and lectin complement pathway in the innate defense against pathogenic *Aspergillus* species. Infect. Immun..

[132] Jepsen C.S., Dubey L.K., Colmorten K.B., Moeller J.B., Hammond M.A., Nielsen O., Schlosser A., Templeton S.P., Sorensen G.L., Holmskov U. (2018). FIBCD1 binds *Aspergillus fumigatus* and regulates lung epithelial response to cell wall components. Front. Immunol..

[133] Khosravi A.R., Alheidary S., Nikaein D., Asghari N. (2018). *Aspergillus fumigatus* conidia stimulate lung epithelial cells (TC-1 JHU-1) to produce IL-12, IFNγ, IL-13 and IL-17 cytokines: Modulatory effect of propolis extract. J. Mycol. Med..

[134] Beisswenger C., Hess C., Bals R. (2012). *Aspergillus fumigatus* conidia induce interferon-β signalling in respiratory epithelial cells. Eur. Respir. J..

[135] Balloy V., Sallenave J.M., Wu Y., Touqui L., Latgé J.P., Si-Tahar M., Chignard M. (2008). *Aspergillus fumigatus*-induced interleukin-8 synthesis by respiratory epithelial cells is controlled by the phosphatidylinositol 3-kinase, p38 MAPK, and ERK1/2 pathways and not by the toll-like receptor-MyD88 pathway. J. Biol. Chem..

[136] Zhang Z., Liu R., Noordhoek J.A., Kauffman H.F. (2005). Interaction of airway epithelial cells (A549) with spores and mycelium of *Aspergillus fumigatus*. J. Infect..

[137] Jhingran A., Kasahara S., Shepardson K.M., Junecko B.A., Heung L.J., Kumasaka D.K., Knoblaugh S.E., Lin X., Kazmierczak B.I., Reinhart T.A. (2015). Compartment-specific and sequential role of MyD88 and CARD9 in chemokine induction and innate defense during respiratory fungal infection. PLoS Pathog..

[138] Okaa U.J., Bertuzzi M., Fortune-Grant R., Thomson D.D., Moyes D.L., Naglik J.R., Bignell E. *Aspergillus fumigatus* drives tissue damage via iterative assaults upon mucosal integrity and immune homeostasis. bioRxiv.

[139] Osherov N. (2012). Interaction of the pathogenic mold *Aspergillus fumigatus* with lung epithelial cells. Front. Microbiol..

[140] Bigot J., Guillot L., Guitard J., Ruffin M., Corvol H., Balloy V., Hennequin C. (2020). Bronchial epithelial cells on the front line to fight lung infection-causing *Aspergillus fumigatus*. Front. Immunol..

[141] Bertuzzi M., Howell G.J. (2021). Single-cell analysis of fungal uptake in cultured airway epithelial cells using differential fluorescent staining and imaging flow cytometry. Methods Mol. Biol..

[142] Chaudhary N., Datta K., Askin F.B., Staab J.F., Marr K.A. (2012). Cystic fibrosis transmembrane conductance regulator regulates epithelial cell response to *Aspergillus* and resultant pulmonary inflammation. Am. J. Respir. Crit. Care Med..

[143] Nemecek J.C., Wüthrich M., Klein B.S. (2006). Global control of dimorphism and virulence in fungi. Science.

[144] Köhler J.R., Casadevall A., Perfect J. (2014). The spectrum of fungi that infects humans. Cold Spring Harb. Perspect. Med..

[145] Sephton-Clark P., Voelz K. (2018). Spore germination of pathogenic filamentous fungi. Adv. Appl. Microbiol..

[146] Hohl T.M., Van Epps H.L., Rivera A., Morgan L.A., Chen P.L., Feldmesser M., Pamer E.G. (2005). *Aspergillus fumigatus* triggers inflammatory responses by stage-specific beta-glucan display. PLoS Pathog..

[147] Steele C., Rapaka R.R., Metz A., Pop S.M., Williams D.L., Gordon S., Kolls J.K., Brown G.D. (2005). The beta-glucan receptor dectin-1 recognizes specific morphologies of *Aspergillus fumigatus*. PLoS Pathog..

[148] Gersuk G.M., Underhill D.M., Zhu L., Marr K.A. (2006). Dectin-1 and TLRs permit macrophages to distinguish between different *Aspergillus fumigatus* cellular states. J. Immunol..

[149] Bozza S., Gaziano R., Spreca A., Bacci A., Montagnoli C., di Francesco P., Romani L. (2002). Dendritic cells transport conidia and hyphae of *Aspergillus fumigatus* from the airways to the draining lymph nodes and initiate disparate Th responses to the fungus. J. Immunol..

[150] Netea M.G., Warris A., Van der Meer J.W., Fenton M.J., Verver-Janssen T.J., Jacobs L.E., Andresen T., Verweij P.E., Kullberg B.J. (2003). *Aspergillus fumigatus* evades immune recognition during germination through loss of toll-like receptor-4-mediated signal transduction. J. Infect. Dis..

[151] Gazendam R.P., van Hamme J.L., Tool A.T., Hoogenboezem M., van den Berg J.M., Prins J.M., Vitkov L., van de Veerdonk F.L., van den Berg T.K., Roos D. (2016). Human neutrophils use different mechanisms to kill *Aspergillus fumigatus* conidia and hyphae: Evidence from phagocyte defects. J. Immunol..

[152] Alekseeva L., Huet D., Féménia F., Mouyna I., Abdelouahab M., Cagna A., Guerrier D., Tichanné-Seltzer V., Baeza-Squiban A., Chermette R. (2009). Inducible expression of beta defensins by human respiratory epithelial cells exposed to *Aspergillus fumigatus* organisms. BMC Microbiol..

[153] Loeffler J., Haddad Z., Bonin M., Romeike N., Mezger M., Schumacher U., Kapp M., Gebhardt F., Grigoleit G.U., Stevanović S. (2009). Interaction analyses of human monocytes co-cultured with different forms of *Aspergillus fumigatus*. J. Med. Microbiol..

[154] Shah A., Kannambath S., Herbst S., Rogers A., Soresi S., Carby M., Reed A., Mostowy S., Fisher M.C., Shaunak S. (2016). Calcineurin orchestrates lateral transfer of *Aspergillus fumigatus* during macrophage cell death. Am. J. Respir. Crit. Care Med..

[155] Levitz S.M., Diamond R.D. (1985). Mechanisms of resistance of *Aspergillus fumigatus* conidia to killing by neutrophils in vitro. J. Infect. Dis..

[156] Ellett F., Jorgensen J., Frydman G.H., Jones C.N., Irimia D. (2017). Neutrophil interactions stimulate evasive hyphal branching by *Aspergillus fumigatus*. PLoS Pathog..

[157] Schaffner A., Douglas H., Braude A.I., Davis C.E. (1983). Killing of *Aspergillus* spores depends on the anatomical source of the macrophage. Infect. Immun..

[158] Cornish E.J., Hurtgen B.J., McInnerney K., Burritt N.L., Taylor R.M., Jarvis J.N., Wang S.Y., Burritt J.B. (2008). Reduced nicotinamide adenine dinucleotide phosphate oxidase-independent resistance to *Aspergillus fumigatus* in alveolar macrophages. J. Immunol..

[159] Grimm M.J., Vethanayagam R.R., Almyroudis N.G., Dennis C.G., Khan A.N., D’Auria A.C., Singel K.L., Davidson B.A., Knight P.R., Blackwell T.S. (2013). Monocyte- and macrophage-targeted NADPH oxidase mediates antifungal host defense and regulation of acute inflammation in mice. J. Immunol..

[160] Botterel F., Gross K., Ibrahim-Granet O., Khoufache K., Escabasse V., Coste A., Cordonnier C., Escudier E., Bretagne S. (2008). Phagocytosis of *Aspergillus fumigatus* conidia by primary nasal epithelial cells in vitro. BMC Microbiol..

[161] Escobar N., Ordonez S.R., Wösten H.A., Haas P.J., de Cock H., Haagsman H.P. (2016). Hide, keep quiet, and keep low: Properties that make *Aspergillus fumigatus* a successful lung pathogen. Front. Microbiol..

[162] Richard N., Marti L., Varrot A., Guillot L., Guitard J., Hennequin C., Imberty A., Corvol H., Chignard M., Balloy V. (2018). Human bronchial epithelial cells inhibit *Aspergillus fumigatus* germination of extracellular conidia via FleA recognition. Sci. Rep..

[163] Araujo R., Rodrigues A.G. (2004). Variability of germinative potential among pathogenic species of *Aspergillus*. J. Clin. Microbiol..

[164] Gresnigt M.S., Becker K.L., Leenders F., Alonso M.F., Wang X., Meis J.F., Bain J.M., Erwig L.P., van de Veerdonk F.L. (2018). Differential kinetics of *Aspergillus nidulans* and *Aspergillus fumigatus* phagocytosis. J. Innate Immun..

[165] Paulussen C., Hallsworth J.E., Álvarez-Pérez S., Nierman W.C., Hamill P.G., Blain D., Rediers H., Lievens B. (2017). Ecology of aspergillosis: Insights into the pathogenic potency of *Aspergillus fumigatus* and some other *Aspergillus* species. Microb. Biotechnol..

[166] Keizer E.M., Valdes I.D., Forn-Cuni G., Klijn E., Meijer A.H., Hillmann F., Wösten H., de Cock H. (2021). Variation of virulence of five *Aspergillus fumigatus* isolates in four different infection models. PLoS ONE.

[167] Caffrey-Carr A.K., Kowalski C.H., Beattie S.R., Blaseg N.A., Upshaw C.R., Thammahong A., Lust H.E., Tang Y.W., Hohl T.M., Cramer R.A. (2017). Interleukin 1α is critical for resistance against highly virulent *Aspergillus fumigatus* isolates. Infect. Immun..

[168] Knox B.P., Blachowicz A., Palmer J.M., Romsdahl J., Huttenlocher A., Wang C.C., Keller N.P., Venkateswaran K. (2016). Characterization of *Aspergillus fumigatus* isolates from air and surfaces of the international space station. mSphere.

[169] Young A.M., Palmer A.E. (2017). Methods to illuminate the role of *Salmonella* effector proteins during infection: A review. Front. Cell. Infect. Microbiol..

[170] Knox B.P., Deng Q., Rood M., Eickhoff J.C., Keller N.P., Huttenlocher A. (2014). Distinct innate immune phagocyte responses to *Aspergillus fumigatus* conidia and hyphae in zebrafish larvae. Eukaryot. Cell.

[171] Knox B.P., Huttenlocher A., Keller N.P. (2017). Real-time visualization of immune cell clearance of *Aspergillus fumigatus* spores and hyphae. Fungal Genet. Biol..

[172] Rosowski E.E., Knox B.P., Archambault L.S., Huttenlocher A., Keller N.P., Wheeler R.T., Davis J.M. (2018). The zebrafish as a model host for invasive fungal infections. J. Fungi.

[173] Koch B., Hajdamowicz N.H., Lagendijk E., Ram A., Meijer A.H. (2019). *Aspergillus fumigatus* establishes infection in zebrafish by germination of phagocytized conidia, while *Aspergillus niger* relies on extracellular germination. Sci. Rep..

[174] Rosowski E.E. (2020). Illuminating macrophage contributions to host-pathogen interactions in vivo: The power of zebrafish. Infect. Immun..

[175] Thrikawala S., Rosowski E.E. (2020). Infection of zebrafish larvae with *Aspergillus* spores for analysis of host-pathogen interactions. J. Vis. Exp. JoVE.

[176] Meeker N.D., Trede N.S. (2008). Immunology and zebrafish: Spawning new models of human disease. Dev. Comp. Immunol..

[177] Hamied A., Alnedawy Q., Correia A., Hacker C., Ramsdale M., Hashimoto H., Kudoh T. (2020). Identification and characterization of highly fluorescent pigment cells in embryos of the Arabian killifish (*Aphanius dispar*). iScience.

